# Plant foods biodiversity in national food composition databases – The Italian case

**DOI:** 10.1017/S1368980026102547

**Published:** 2026-04-29

**Authors:** Silvia Lisciani, Elisabetta Toti, Emanuela Camilli, Stefania Marconi, Catherine Leclercq

**Affiliations:** Consiglio per la ricerca in agricoltura e l’analisi dell’economia agraria (CREA), Rome, Italy

**Keywords:** Biodiversity, Food composition database, Plant-based foods, Dietary species richness

## Abstract

**Objective:**

To assess the biodiversity richness of plant foods of the Italian Food Composition Database (IFCDB) at the species and below the species level and its evolution over time.

**Design and Setting:**

The biodiversity richness of plant foods in the IFCDB was assessed by counting the number of species and by identifying and categorising biodiverse plant foods, i.e. foods described below the species level (subspecies, variety and cultivar) as well as wild and neglected and underutilised species. This assessment was also performed with the FAO Biodiversity Indicator.

**Participants:**

This study analysed the current IFCDB that contains 900 records of food items, with 80 % of data derived from analytical determinations.

**Results:**

The 2019 IFCDB’s edition includes 114 plant species; among thirty two of them, one or more biodiverse foods were identified for a total of eighty-six records, corresponding to 21 % of the plant foods recorded. This marks a substantial increase from the 2000’s edition, which included 112 plant species and forty-eight biodiverse foods, corresponding to 16 % of the plant foods recorded.

**Conclusions:**

The IFCDB demonstrates progress in integrating plant food biodiversity, crucial for promoting sustainable diets and, consequently, sustainable food systems. Enhanced access to food composition data of biodiverse plant foods is required for the development and labelling of biodiverse processed plant foods and to increase the biodiversity richness of menus in community catering. This study may stimulate efforts in assessing and enhancing biodiversity richness of food composition tables in other countries.

Food Composition Databases (FCDB) are largely used in public health nutrition as a key tool for promoting healthier diets. Their potential for fostering more sustainable food systems, including greater biodiversity, remains underexploited. Biodiversity, as defined in 1992 by the Convention on Biological Diversity, refers to the variability among living organisms from all sources, including terrestrial, marine and other aquatic ecosystems, as well as the ecological complexes of which they are a part^(1)^.

Plant organisms, including those used for human food consumption, can be identified through taxonomic nomenclature, i.e. can be identified by genus and species and lower taxonomic levels such as subspecies, variety or cultivar. A ‘subspecies’ (ssp) refers to a species’ subgroup which aggregates varieties naturally developed in a specific geographical area. It is the case of durum wheat also called pasta wheat (*Triticum turgidum* ssp. *durum* where the first word, *Triticum,* is the genus whereas the second word *turgidum* defines the species). A ‘variety’ (var.) refers to a species’ subgroup with specific morphological or genetic traits developed through natural selection, which are of interest for cultivation^(2)^. It is the case of bell pepper (*Capsicum annuum* var. *Grossum).* A ‘cultivar’ (cv), short for cultivated variety, refers to a species’ subgroup intentionally selected and maintained by human intervention for desired characteristics, such as size, flavour or nutrient density^(2)^. It is the case of Venere rice (*Oryza sativa* cv *Venere*), which was specifically developed by humans mainly for its organoleptic properties.

FAO defines *biodiverse foods* as foods identified below the species level (e.g. variety, cultivar, breed and market type) alongside wild and neglected and underutilised foods (NUS) indicated at the species level^(2)^. NUS are identified as ‘species with underexploited potential for contributing to food security, health and nutrition, income generation, and environmental services’^(3,4)^. These species are sometimes named orphans, underdeveloped, lost, new, novel, promising, alternative, local, traditional, niche or biodiverse. What characterises them is that they are not part of the agricultural mainstream crops. Considering that the concept of neglected and underutilised species foods is dynamic, the status of a species can change in relation to geography and time^(5)^. Foods are considered NUS by FAO only if present in the ‘Reference list of underutilised foods for food biodiversity’ available on the INFOODS website^(4)^. FAO considers wild species those foods which thrive in their natural habitat without human intervention^(2)^.

Biodiversity loss, along with climate change, is recognised as a major threat to life on earth^(6)^.

The consumption of foods with greater biodiversity may stimulate a demand-driven increase in crops with more genetic variability, thereby promoting more biodiversity in general and among the living organisms involved in agricultural food production^(7,8)^. To date, four genera/species -sugarcane (*Saccharum officinarum*), maize (*Zea mays*), wheat (*Triticum* genus) and rice (*Oryza* genus) account for about half of global primary crop production. Furthermore, 90 % of the world’s energy intake comes from just fifteen genera/species^(5,9)^. *Triticum aestivum* (commonly named ‘bread wheat’) accounts for approximately 90 % of global wheat production and *T. turgidum* ssp. *durum* (commonly called ‘pasta wheat’), contributes another 5–10 %^(5,9)^. Similarly, the rice species *O. sativa* is the main staple food for nearly half the world’s population, accounting for 21 % of global energy intake^(10)^. High-yielding crops – in particular soyabeans, corn, wheat, sugarcane and palm fruits are largely used in the preparation of ultra-processed foods through sequential processes, starting from the fractionation of crops into starches, fibers, sugars, oils and fats and proteins^(11,12)^. Promoting biodiversity, therefore, means shifting away from diets based on a very limited number of staple plant foods.

In this article, the term ‘biodiverse plant foods’ refers to plant foods identified below the species level (subspecies, cultivar, variety) in addition to wild and NUS plants foods.

FAO advocates for the collection of high-quality food consumption and food composition data identified below the species level^(5,13)^, considering it a necessary step toward promoting more biodiverse diets.

Indeed, fostering crop biodiversity can have a significant positive effect on the overall sustainability of diets, encompassing socio-economic, nutritional and environmental aspects^(7)^.

National FCDB are comprehensive resources that provide detailed information on the energy content, macronutrients, micronutrients and bioactive components of foods^(14,15)^. The foods included in FCDB vary by country, as they must encompass a broad range of foods reflecting the typical diet of the population, with particular attention to those that contribute significantly to nutrient intake. In many countries, evolving consumer preferences, the increased reliance on processed products, the emergence of novel foods and the expansion of global trade necessitate regular updates of FCDB to include compositional data for an ever-broader spectrum of foods^(16,17)^. Figure 1 illustrates the diverse stakeholders who utilise food composition databases and could potentially benefit from more biodiversity richness in these data.

**Figure 1.**
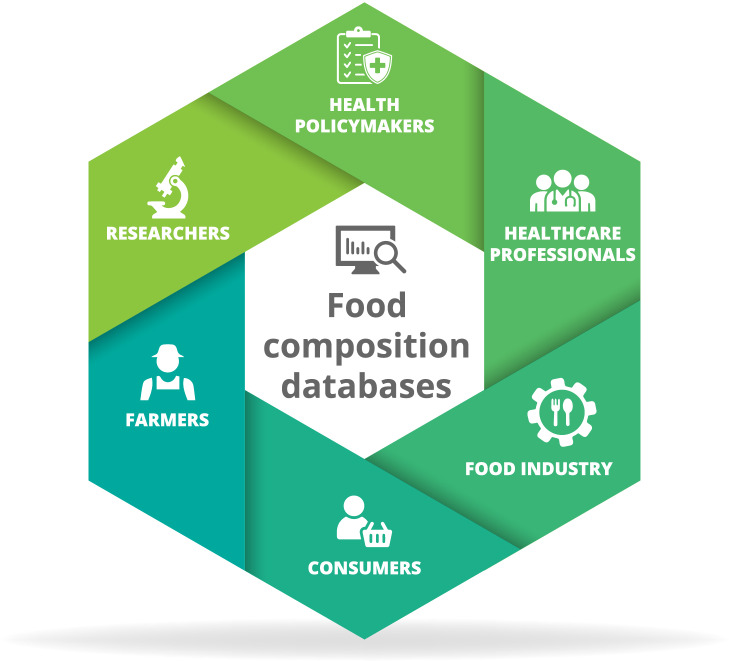
Stakeholders who use Food Composition Databases.

Despite the growing recognition of its importance, data on biodiversity in food composition remain scarce. Most national FCDB describe foods primarily at the species level, often neglecting to identify subspecies/varieties/cultivars for plant foods or breeds for animal foods. For instance, national databases typically provide a generic entry for ‘Tomato’ (*Solanum lycopersicum*), often failing to account for the significant phenotypic and compositional diversity found between specific varieties or cultivars, such as ‘San Marzano’ tomato (*S. lycopersicum* cv ‘San Marzano’) or cherry tomato (*S. lycopersicum* var. cerasiforme). This limitation extends to wild and NUS species, which are frequently lacking^(5)^. FAO has recognised that increased efforts and resources are essential to analyse and disseminate the nutrient profiles of wild and NUS foods, thereby helping countries promote local species and preserve their ecosystems^(5)^. The micronutrient superiority of some lesser-known cultivars and wild varieties over other extensively utilised cultivars has been observed in some low and middle-income countries^(8,18)^. Progress towards greater biodiversity richness in FCDB at global level has been very slow in the last ten years. The nutrient composition of biodiverse foods remains largely unknown, which limits both the awareness of their importance and their use by farmers and consumers^(3,19)^. A recent initiative of the FCDB’s crosscutting ‘Biodiversity for Food and Nutrition’ project is aimed at promoting 189 under utilised species based on their specific nutritional composition^(7)^. Furthermore, the FAO/INFOODS biodiversity database^(13)^ collects analytical data on food biodiversity at the variety/cultivar, breed and species levels.

Parallel to the growing interest of nutritionists in food biodiversity, metrics of the biodiversity richness of food consumption and food composition databases have been developed. For example, the dietary species richness (DSR) metric counts the number of unique edible species in food consumption databases to assess the biodiversity richness of diets^(20,21)^. Recent research suggests that these metrics are key to addressing public health challenges, and DSR was shown to be far more than a descriptive tool. DSR is a critical predictor of nutritional adequacy, clinical outcomes and it inversely correlates to all-cause mortality^(20)^. Biodiversity has become increasingly important in studies on diet quality because conventional metrics of dietary diversity fail to capture the critical nutritional variability driven by biodiversity richness and dissimilarity within food groups^(21)^. The FAO’s ‘Food Composition Indicator for Biodiversity and Nutrition’ – hereafter referred to as the ‘FAO Biodiversity Indicator’ – counts the number of biodiverse foods in FCDB to assess their potential to promote biodiverse foods based on their nutrient composition^(22)^.

The present article focuses only on plant foods reported in IFCDB. Italy is characterised by a high level of biodiversity of plants in general and of plant foods in particular^(23)^. The first collection of Italian food composition data was published in 1946 and contained only 198 food items with data on proteins, lipids, carbohydrates and calories. These were followed over the years by seven printed updates, leading to the latest digital edition in 2019^(24)^.

## Aim of the study

The aim of this study is to assess the biodiversity richness of plant foods of the Italian Food Composition Database (IFCDB) at the species and below the species level, and its evolution over time.

## Methods

The IFCDB comprises 900 food items categorised into twenty distinct groups. Each record is identified by a unique alphanumeric code, its Italian and English common names and the Latin scientific name (hereafter referred to as the *scientific name*). The biodiversity richness of plant foods reported in the IFCDB was assessed at two levels: species and below the species. The total number of plant food species was counted with the same methodology used for the DSR metric for food consumption data^(20,21)^. This count was possible because the scientific name is reported at least at the genus and species level for all foods in the IFCDB.

Biodiversity was then assessed by identifying and categorising records that describe biodiverse plant foods, i.e. wild species and NUS species, and foods described below the species level (subspecies, varieties and cultivars), described in any way (e.g. by Italian name, phenotypic characteristics). In fact, many foods lack this level of detail in their scientific description, with such distinctions appearing only in the Italian name (e.g. escarole; endive). Similarly, foods for which information on biodiversity could be retrieved through more generic descriptors (such as shape and colour) were also considered, treating these morphological characteristics as an indication of biodiversity. For food items reported without a scientific name below the species level but with variety and cultivar information in the Italian name, this information was confirmed using external botanical databases, and the appropriate scientific name was assigned to them^(25–27)^. The biodiversity of the IFCDB was also assessed using the FAO Biodiversity Indicator^(5)^.

## Results

### Main characteristics of the Italian Food Composition Database

The IFCDB is produced, managed and published by a team of food composition experts from the Council for Agricultural Research and Economics (CREA) Food and Nutrition Centre. The most recent update was released in 2019 and is available online at: https://www.crea.gov.it/-/tabella-di-composizione-degli-alimenti ^(24)^. Compared with the previous version^(28)^, this update includes analytical composition data for a further 148 food items. In addition, the nutrient analysis of some foods was re-performed due to changes in their composition over time. For instance, the IFCDB contains new analytical data for pasta, as the type of wheat used and the production technologies for this Italian staple food have changed, altering its composition. The protein content, for example, rose from 10·9 g/100 g to 13·5 g/100 g in the current version. Sampling procedures, analytical determinations, documentation and data compilation are carried out according to the current international guidelines and standards of EuroFIR AISBL^(29)^ and AOAC^(30)^. The current version of the Italian FCDB contains 900 items and reports a total of 120 components. These include not only energy, water, macro- and micronutrients, fatty acids and amino acids but also other components such as phytic acid, organic acids (malic, caffeic and citric acid), antioxidant activity index and bioactive compounds.

Overall, about 80 % of the data are derived from analytical determination conducted in dedicated laboratories on foods available on the Italian market, ensuring a reliable analytical origin. About 2 % consists of calculated or estimated data from comparable foods. Other data were derived from a careful bibliographic selection, mainly from Italian sources^(24)^.

### Biodiversity in the IFCDB

Historically, the IFCDB’s chemical analyses relied on blended samples of widely consumed food items, which were identified through national food consumption surveys. In some cases, different varieties and cultivars of the same food were collected and blended, resulting in a single analytical value that failed to capture food composition differences related to biodiversity. Since the 2000s changes in Italian dietary habits, including an increased consumption of foods from other countries, have prompted the need for more detailed studies on food composition that reflect this greater dietary variety. As a result, recent efforts have focused on analysing foods below the species level, including varieties, cultivars and wild or NUS plant foods. This shift is also supported by the national food policy, which has been implemented to promote Italian agricultural biodiversity. Indeed, Italy is the EU country with the highest number of products with EU geographical certification labels with 150 protected geographical indication (PGI) and 174 protected designation of origin (PDO), in addition to the 5717 products with traditional agri-food products (PAT) Italian geographical certification label^(31,32)^. These labels testify the strong connection between the specific qualities of foods and the specific territory where they are produced. Some PDO, PGI and PAT agricultural products are promoted as healthy based on their specific cocktail or concentration of nutrients and bioactive substances. A total of nine specific varieties and cultivars with geographical certification label were sampled, analysed and added to the last version of IFCDB thanks to *ad hoc* funding from the Italian Ministry of Agricultural Policies, Food Sovereignty and Forests^(33)^ for inclusion in the IFCDB, even if their consumption volumes are low at national level. Thus, the increased biodiversity richness of the IFCDBs is in line with the growing interest in food biodiversity that has emerged in recent years.

The current edition of the IFCDB includes 114 plant species, distributed across various food groups as presented in Table 1.

**Table 1.** Distribution of plant food species in the Italian Food Composition Database by food group

Food group	Number of species^*^
Fruits	41
Vegetables and tubers	36
Cereals and derivatives	11
Nuts	11
Legumes	8
Oils and oilseeds	12
Various products	3

* The sum of the number of species in each food group is greater than 114 because some plant food species are present both as food consumed as such and as an oil extract. An example is coconut, which is present both in the fruit category and as coconut oil. Food groups are based primarily on nutritional and usage characteristics rather than botanical classification.

It includes eighty-six biodiverse plant foods, i.e. plant foods described below the species level (*n* 78), NUS (*n* 1) or wild (*n* 7). Biodiverse plant foods represent 21 % of the plant foods recorded. They belong to thirty-two plant food species that represent 28 % of the species listed. The types of biodiversity description below the species level include (i) foods with cultivar/variety level in the scientific name, (ii) foods with the cultivar or variety reported only in the Italian name and (iii) varieties and cultivars indicated in generic manner: by colour, by use (e.g. ‘salad-type’) (Table 2).

**Table 2.** Biodiversity description of plant foods in the Italian Food Composition Database

Biodiversity description	No.	Example (English and Italian name)
Subspecies/variety/cultivar reported in the scientific name	34	Cauliflower – Cavolfiore (*Brassica oleracea* var. *botrytis*)
Subspecies/variety/cultivar indicated only in the Italian name^*^	31	Venere rice – Riso venere (*Oryza sativa* cv. *venere*)
Subspecies/variety/cultivar described generically (e.g. by colour or common use)	13	Light green Zucchini – Zucchine chiare (*Cucurbita pepo*); Salad tomato – Pomodoro da insalata (*Solanum lycopersicum*)
Wild species	7	Wild Asparagus – Asparago selvatico (*Asparagus acutifolius*); Wild chicory – Cicoria selvatica (*Cichorium intybus*); Wild Blackberry – Mora di rovo (*Rubus fruticosus*)
Underutilised food species	1	Cowpea – Fagiolo dall’occhio (*Vigna unguiculata*)

* Cultivar indicated only in the Italian common name, without specific scientific classification.

**Figure 2.**
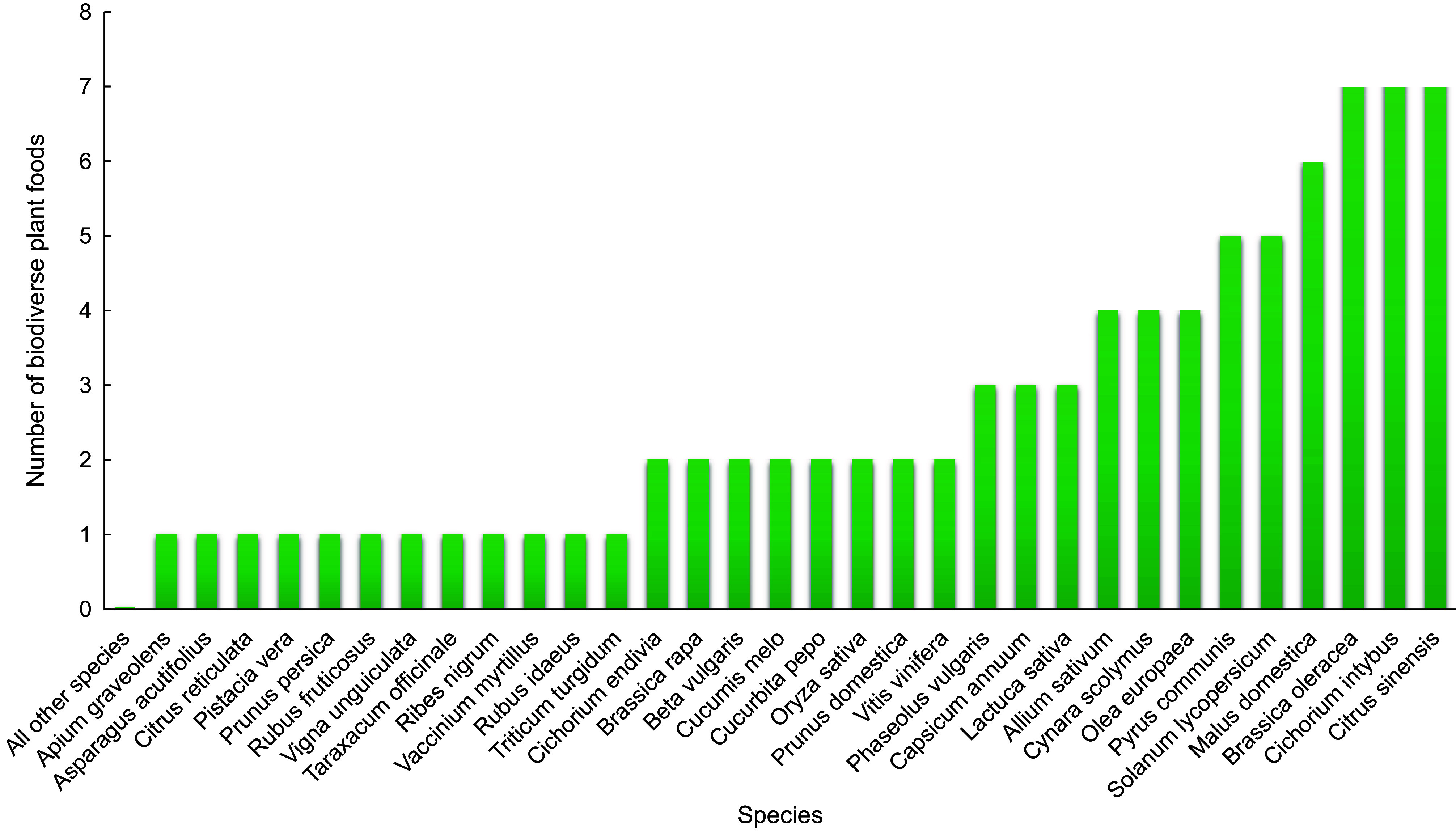
Number of biodiverse plant foods described in the Italian Food Composition Database. Biodiverse plant foods refer to plant foods described below the species level (subspecies, cultivar, variety) in addition to wild and neglected and underutilised foods (NUS) plants species.

Only one NUS food, *Vigna unguiculata* (cowpea), is present in the IFCDB. It is a food known for its environmental sustainability, domesticated in Africa, and commonly grown also in Asia and Latin America. Once popular in Italy, particularly in the Campania region, its cultivation declined due to the introduction of higher-yielding Common Beans (*Phaseolus vulgaris)*. By the 1980s, cowpea was primarily grown in family gardens; it is now cultivated in limited areas of Campania and Apulia^(34,35)^. Figure 2 shows the number of biodiverse plant foods for each species, ranging from one to seven. Some species, such as *Apium graveolens, Asparagus acutifolius, Citrus reticulata, Pistacia vera* and *Prunus persica* exhibit only one food item identified. In contrast, each of the species of *Brassica oleracea, Cichorium intybus* and *Citrus sinensis* exhibits seven food items described below the species level.

Among the eighty-six biodiverse plant foods, the majority are vegetables (44), followed by fruits (34), legumes (4), cereals (3) and one nut species (pistachio- *Pistacia vera*).

Sixteen of these foods have obtained over time a geographical certification label (PDO, PGI or PAT) (Table 3).

**Table 3.** Plant foods with geographic certification labels present in Italian Food Composition Database

PAT^*^	PDO^†^	PGI^‡^
Piacentino white garlic, aglio bianco Piacentino (*Allium sativum*)	Pistachio from Bronte, Pistacchio di Bronte (*Pistacia vera* cv. *Bianca*)	Roman Globe Artichoke, Carciofo Romanesco (*Cynara scolymus* cv. *Campagnano*)
Castelliri red garlic, aglio rosso di Castelliri (*Allium sativum*)	Nocellara del Belice olive, oliva di Nocellara del Belice (*Olea europaea* cv. *Nocellara del Belice*)	Radicchio Rosso di Treviso Late Variety, Radicchio Rosso di Treviso varietà tardiva (*Cichorium intybus*)
Proceno red garlic, aglio rosso di Proceno (*Allium sativum*)	Sardinian spiny Globe Artichokes, – Carciofo spinoso sardo *(Cynara scolymus)*	Radicchio Rosso di Treviso Early Variety, Radicchio Rosso di Treviso varietà precoce (*Cichorium intybus*)
Sulmona red garlic, aglio rosso di Sulmona (*Allium sativum*)	San Marzano tomato – Pomodoro San Marzano (*Solanum lycopersicum*)	Variegated Radicchio of Castelfranco, radicchio variegato di Castelfranco (*Cichorium intybus*)
		Tarocco orange – Arancia Tarocco (*Citrus sinensis* var. *Tarocco*)^§^
		Moro orange – Arancia Moro (*Citrus sinensis* var*. Moro*)^§^
		Sanguinello orange – Arancia Sanguinello (*Citrus sinensis* var. *Sanguinello*)^§^
		Annurca Apple – Mela Annurca (*Malus domestica*)

* Traditional agri-food products (PAT): This geographical certification label is regulated by the Italian Ministry of Agriculture, Food Sovereignty and Forests^(32)^. It protects products ‘obtained with processing, conservation and seasoning methods consolidated over time, homogeneous throughout the territory concerned, according to traditional rules, for a period of no less than twenty-five years’.

† Protected Designation of Origin (PDO): This geographical certification label is regulated at EU level. Every part of the production, processing and preparation process must take place in the specific region^(31)^.

‡ Protected Geographical Indication (PGI): This geographical certification label is regulated at EU level. For these products, at least one of the stages of production, processing or preparation takes place in the region regulated at EU level^(31)^.

§ The three varieties of orange *Moro*, *Sanguinello* and *Tarocco* are included in the list of PGI under the single designation ‘Arancia Rossa di Sicilia’ (Red Orange of Sicily), a name reserved exclusively for these varieties^(31)^.

Of the eighty-six biodiverse plant foods, a total of nineteen (two common beans, two rice and fifteen vegetables varieties/cultivars) have been analysed not only in their raw form but also after one or more cooking methods or processing (including canning). These twenty-four duplicate records were counted only once as they represent different forms of the same food. On the contrary, coral bean (*Phaseolus vulgaris*) is present in the IFDCB only as cooked.

### Application of the FAO Biodiversity Indicator

Overall, fifty-two plant foods present in the current IFCDB (13 % of the plant foods listed) are considered biodiverse according to the FAO Biodiversity Indicator. The criteria for inclusion and exclusion as reported by Stadlmayr^(22)^ are illustrated in Table 4 with examples taken from the IFCDB.

**Table 4.** Criteria for inclusion and exclusion of foods based on the FAO Biodiversity Indicator for food composition

Foods included	Example	Foods excluded	Example
Foods at cultivar/variety/breed level for common and imported foods, preferably with scientific name	Roman Globe Artichokes – Carciofo romanesco *(Cynara scolymus,* cv. *Campagnano)*	Common or imported foods described only at species level, even if other specifications are given such as: 1. region; 2. country; 3. season; 4. colour as part of the food name or as indication of processing; 5. shape; 6. species name is followed by author, which should not be confused with the cultivar/variety/breed name; 7. local name.	Red radicchio; Radicchio rosso, (*Cichorium intybus);* Green Globe Artichokes early variety – Carciofo verde, precoce *(Cynara scolymus);* Black Olives – Olive nere (*Olea europea*); Salad tomato – Pomodori da insalata (*Solanum lycopersicum*)
For those foods counting for the indicator: – different parts of plants (e.g. leaf, root, flower, stem and fruit) and animal; – different stages; – only raw foods; except if just the cooked form of this food is available	Oranges Moro – Arance Moro *(Citrus sinensis* var*. Moro)* Venere rice – Riso venere *(Oryza sativa)* Coral bean – fagiolini corallo (*Phaseolus vulgaris*), cooked	Common or imported foods described only with local name	not present in IFCDB^*^
Ingredients, if they meet the criteria, used in: – recipes or processed foods; – non-packaged form of botanical supplements/extracts (including beverages)	Not considered in the study	Foods with unspecific name	Not present in IFCDB^*^
Foods with the number of cultivars/varieties/breeds per species even if not described by taxonomic or local name	Winter melon – Melone d’inverno *(Cucumis melo* var*. Reticulatus* and var. *Cantalupensis)*	Local name in addition to English/Spanish/French name seeming to be the translation of the food (e.g. not indicative of variety/cultivar/breed)	Not present in IFCDB^*^
Wild and/or underutilised foods only described at genus/species level and/or with local name	Blackberry, wild – Mora di rovo (*Rubus fruticosus*)	Processed foods or recipes	Cauliflowers cooked in microwave. Cavolfiore cotto al microonde (*Brassica oleracea* var*. Botrytis*)
The underutilised foods must be recorded in the ‘list of underutilised species counting for food biodiversity’	Not present in IFCDB^*^		
A local name in addition to an English/Spanish/French or taxonomic name if it is indicative for a variety/cultivar/breed	Not present in IFCDB^*^	supplements, and plant or animal extracts in packaged form	Not present in IFCDB^*^
Colour and/or shape describe the variety/cultivar/breed	Radicchio Rosso di Treviso Late Variety, Radicchio Rosso di Treviso varietà tardiva (*Cichorium intybus*)	Fortified foods	Not present in IFCDB^*^
Taxonomic varieties commonly considered as a species, if described with additional cultivar name^†^	Green bell pepper – Peperone verde (*Capsicum annuum* var*. Grossum* cv*. Yolo wonder*)	Taxonomic varieties commonly considered as a species, if described without an additional cultivar name^†^	Cabbage – Cavolo *(Brassica oleracea,* var*. Capitata)*
GM foods	Not present in IFCDB^*^		

* Italian Food Composition Database.

† A key inclusion criterion for the FAO indicator requires that foods be identified at the cultivar, variety or breed level. The FAO provides an exception for varieties that are phenotypically so different from each other that they are mistakenly considered distinct species. This is the case for the different varieties of *Brassica oleracea.*

It immediately appears that among the eighty-six biodiverse plant foods, not all were counted in the FAO Biodiversity Indicator due to the exclusion criteria. For example, thirteen foods were excluded because the information below the species was only based on generic indications, such as colour or season. In terms of NUS, the only food considered as NUS in Italy (cowpea) was not counted in the FAO’s biodiversity indicator^(5)^, since it does not appear in the FAO list of NUS for Italy^(4)^.

The biodiversity richness of the IFCDB was also assessed by counting the number of food components – nutrients and non-nutrients – analysed for biodiverse plant foods, as suggested by FAO. Among the fifty-two foods of the IFCDB meeting the FAO biodiversity criteria, fifteen foods were characterised by over thirty components and thirty-seven foods by 10 to 30^(5)^.

### Time trends in the biodiversity richness of the IFCDB

Comparison of the biodiversity richness of the 2019 IFCDB edition with that of the 2000’s paper edition^(28)^, is noteworthy. In the 2000’s edition, food items are described by their Italian names and scientific name with composition data for the following components: energy, vitamins, minerals, lipids and amino acids profiles. The total number of food items listed increased from 680 in the 2000’s edition to 900 in the 2019’s edition, whereas plant foods increased from 307 to 412. Additionally, the number of plant food species increased from 112 to 114, and the number of biodiverse plant foods increased from 48 (16 % of plant foods) to 86 (21 %). Among the 48 biodiverse plant foods of the 2000’s edition, twenty nine are described as such in the Italian name and/or in scientific name, twelve in their generic characteristics (e.g. color, shape), seven are wild and one is a NUS (*Vigna unguiculata*). Foods defined as biodiverse, according to the FAO Biodiversity Indicator criteria, also increased from nineteen in the 2000’s IFCDB’s edition (6 % of all plant foods) to 52 (13 % of all plant foods) in the current IFCDB.

## Discussion

An increased access to food composition data of biodiverse foods was underlined by FAO as crucial for promoting both the consumption of biodiverse foods and the production of biodiverse crops^(19)^. Databases with specific composition data of biodiverse plant foods may help to increase the number of varieties/cultivars consumed at population level by facilitating:

- The use of a larger number of biodiverse plant foods in processed foods. In fact, specific composition data are needed for product development, nutritional labelling and reformulation^(15,36)^.
- The inclusion of a larger number of biodiverse plant foods in community catering menus (schools, workplaces, retirement homes and hospitals) and in planned diets for patients. Indeed, specific composition data must be present in the databases used by dietitians to plan menus and personalised diets with adequate nutrient content.

Increasing the proportion of local sourcing of biodiverse foods in school meals has been suggested as an effective way to support local farmers who cultivate more biodiverse plant foods and less mainstream plant foods. Indeed, the volumes of food distributed in community catering tend to be high and regular. Local farmers may thus have more secure market opportunities^(37)^ and could more easily plan the production of biodiverse annual crops such as cereals, vegetables, legumes and tubers. Upscaling these initiatives is challenging due to the conflict with the interests of powerful monocrop agriculture and food processing companies which maintain the status quo^(11)^. However, it has successfully been applied at large scale in the Brazil’s national school feeding program which provides meals to more than 40 million school children and mandates direct procurement from family farmers. Family farming in Brazil is closely linked to diversified and agroecological food production systems that enhance biodiversity^(8)^. Thus, public procurement programs can help to stimulate local biodiverse agriculture for food production.

Once acceptability by catering users is ensured, the inclusion of more biodiverse foods makes meals more enjoyable and varied, thus stimulating appetite which may be particularly important in children, elderly and hospitalised patients^(37,38)^. Biodiverse foods may also improve adherence to personalised diets, which often tend to be monotonous. A further positive aspect of increasing the consumption of biodiverse foods is that subspecies/varieties/cultivars provide a varied cocktail of nutrients and bioactive substances thus contributing to nutrient adequacy of the diet and to its preventive potential towards non-communicable disease, in line with dietary guidelines.

### Quality of Italian Food Composition Database

The percentage of analytical values in the FCDB is a quality indicator. Indeed, data obtained through direct analytical methods, where stringent quality control and precise sampling techniques are documented, are considered superior to data from published sources or calculations^(39)^. In the case of the current IFCDB, this percentage reaches 80 %.

### Assessment of biodiversity of other databases

This is the first analysis of the biodiversity richness of the IFCDB. In the European region, no assessment of biodiversity richness of national FCDBs has yet been performed. For comparative purpose, the information of biodiverse food items was explored in two prominent European FCDB – the French^(40)^ and the UK’s McCance and Widdowson’s^(41)^. Both integrate analytical, bibliographic selection and calculated data, but a key limitation is their lack of scientific names, which makes the identification of foods below the species level difficult.

The French database has over 3000 food items and occasionally documents below species information when a common name corresponds to a specific variety (e.g. Brussels sprouts and cauliflower). For some fruits, such as apples (Pink Lady, Gala) and pears (Conference, Williams), the varietal name is made explicit. Few entries are geographically specific, such as Martinique pumpkins (Phoenix variety, or more generic ‘local variety’).

The different categories below the species information provided in the UK database are the same as the IFCDB ones. Some common names explicit the varietal names (e.g. Bramley apple, Hass avocado). In other cases, an average of multiple varieties (e.g. average for Cantaloupe, Galia and Honeydew melons) is reported under a single entry. Some below the species level description is provided by the color (e.g. pale chicory, yellow potato) but without any variety/cultivar name. Analytical databases are known to be expensive in terms of cost and time, and it is often difficult to distinguish between compiled and analytical data. To overcome this problem, the FAO/INFOODS Analytical Food Composition Database^(42)^ provides a global compendium containing only scrutinised analytical data from worldwide databases. Each food entry includes the full bibliographic reference, the food’s name in English and its scientific name.

An important effort of post harmonisation of EU food consumption and food composition databases was performed with the use of FoodEx2, the standardised food classification and description system developed by the European Food Safety Authority^(43)^. FCDB from seven countries have been categorised with FoodEx2 and are available at https://www.efsa.europa.eu/en/data-report/food-composition-data ^(43)^. By mid-2026, EFSA will publish a more comprehensive FCDB^(44)^. The use of FoodEx2 to explore the biodiversity richness of these FCDB is still to be performed. The scientific names of plant foods can be extracted from the FoodEx2 standardised descriptors for raw agricultural products (e.g. durum wheat) and as implicit descriptors of derivatives (e.g. durum wheat flour)^(43)^.

FAO created a ‘Food Composition Database for Biodiversity’, which reports composition data of biodiverse foods. These data were retrieved from the literature and underwent a rigorous quality scrutiny before being inserted. The first version was published in 2010 and contained 2401 entries, *v*. 7953 (of which 4897 plant foods items) in the 2017 version^(13)^. It has not been updated since then. The scientific names of cultivars and varieties are not available for all entries due to the difficulty in retrieving this information and in distinguishing a cultivar from a variety^(13)^. However, the ‘Food Composition Database for Biodiversity’ provides a very comprehensive list of biodiverse plant foods consumed worldwide. The development of databases of analytical values of this dimension at country level is not feasible. Thus, a prioritisation process is necessary in each country to select key plant foods for chemical analysis.

### Limitations of the present study

A limitation of this study is the uncertainty in taxonomic nomenclature (subspecies/varieties/cultivars) for thirteen foods in the current IFCDB. For these foods, a generic description is provided in addition to the name of the species (e.g. green *v*. dark green zucchini varieties) without the exact scientific name or Italian name of a variety/cultivar. In other cases, taxonomic assignments are jeopardised by the sampling methods used in the past which focused on providing an estimate of the average composition of different varieties of common species rather than aiming on biodiversity.

Thus, some food items, described or not below the species level in the scientific name, refer to a ‘mix’ of several varieties or cultivars with similar physical characteristics. For example, for bell peppers (*Capsicum annuum, var. grossum*), the composition data for the yellow pepper refers to a pool of two cultivars (*Quadrato d’Asti, Corno di bue*), while the red pepper data come from three cultivars (*Topepo, Quadrato d’Asti, Corno di bue*). Similarly, the winter melon (*Cucumis melo*) comes from the sampling of two different varieties (*var. reticulatus and var*. *cantalupensis*).

Differences in the composition of the different subspecies/varieties/cultivars of a species should not be overinterpreted. Indeed, soil characteristics, season, agronomic techniques, storage conditions, ripeness and water content also determine the composition of the samples collected for analysis. Food composition variability reflects inherent (e.g. genetic), environmental (e.g. climate and temperature), processing (e.g. cooking and preservation methods) and analytical (e.g. sampling designs and analytical methods) factors. Some food components are more variable than others because they may be susceptible to loss by heat, light and processing^(19)^.

This issue is partly overcome when food composition analysis is performed on blended samples of each variety from different geographical areas or suppliers/sellers to obtain the most representative data. In the IFCDB the analysis of some biodiverse foods was performed on a few samples from a unique location. For example, the Nerina tomato was studied at two ripeness stages (green and red), and the sampling was conducted with organic products from a unique Latium farm. In other cases, sampling was more extensive. Two types of olives in brine, Nocellara del Belice (PDO) and Giarraffa, were sampled from five different Sicilian farms. For Treviso red radicchio (PGI), both late and early varieties were sampled from four farms over two consecutive years; Castelfranco variegated radicchio (PGI) was obtained from three producers; Bronte pistachio (PDO) was sampled from two different Sicilian areas at two altitudes (< 600 m and > 600 m). For garlic, the varieties Rosso di Castelliri, Bianco Piacentino, Rosso di Sulmona and Rosso di Proceno were all produced on three different plots in two Lazio areas (Viterbo and Alvito) using the same agronomical techniques^(33)^.

### Dietary considerations

The increased consumption of some subspecies/varieties/cultivars with respect to mainstream plant foods of the same species may enhance dietary adequacy in a population by increasing the intake of key nutrients. This is the case with red bananas (cultivar of the wild banana *Musa acuminata*) in low-income countries where bananas are a staple food. Promoting red bananas is crucial for preventing widespread vitamin A deficiency due to the enormous difference in their carotenoid content. A 1000-fold difference in *β*-carotene was observed between some cultivars and the mainstream cavendish banana^(19)^.

In general, composition differences between different varieties of staple foods are particularly interesting because these foods are consumed in large daily portions by most of the population. Conversely, due to the very small amounts consumed, the specific composition of spices, herbs and condiments like garlic has little impact on dietary adequacy.

Wheat is a staple food in Italy. Ancient wheat, such as einkorn, emmer and Khorasan, unchanged over the last centuries, has been reintroduced recently in the Italian market. Several studies have analysed the protein and mineral content of a few modern and ancient wheats. While some of these studies are limited by sample size and inconsistent results, it is a general finding that ancient wheats possess higher levels of certain bioactive compounds, like lutein, compared with modern bread wheat, which was selectively bred for traits like a whiter grain colour^(45)^. Ancient wheat also tends to be richer in proteins, lipids and minerals such as K, Mg, Ca and and Fe, while modern bread wheat shows higher levels of P, Zn, and Cu^(46)^. Another Italian staple food is rice. Thousands of rice varieties are cultivated worldwide; they all belong to two species: *O. sativa* and *Oryza glaberrima*. *O. sativa* is cultivated and consumed in Italy. Promoting the consumption of more varieties of rice in Italy could be of interest from a nutritional point of view. Indeed, the different varieties of these two species were shown to have distinct nutritional profiles, particularly in protein and mineral content. A review by Kennedy and Burlingame^(47)^, based on analysis from the IRRI International Rice Commission laboratory evidenced that *O. sativa* varieties have protein levels ranging from 4·5 % to 15·9 %, with an average of 8·8 %, while *Oryza glaberrima* varieties range from 10·2 % to 15·9 %, with a higher average of 13·6 %. The mineral content analysis also revealed significant variations in Fe (from 0·70 to 6·35 mg/100 g on a DM basis) and Zn (from 0·79 to 5·89 mg/100 g on a DM basis) among different rice varieties.

Comparisons between different varieties of certain species in the IFCDB have shown significant differences in certain components. For example, some varieties of the *Brassica oleracea* species show substantial variations in total dietary fiber content ranging from 1 g/100 g (variety *Capitata*) to 5 g/100 g (variety *Gemmifera*)^(24)^. The existence of these differences highlights the need for more analytical determinations of the varieties of other widely consumed foods.

### Consideration of the FAO Biodiversity Indicator

The FAO Biodiversity Indicator showed some limitations for its use in the Italian case. As for NUS, it relies on a list which was not updated since 2015 and which underrepresents NUS from high-income countries. Thus, none of Italy’s niche plant foods are included in the INFOODS list^(4)^.

Among the eighty-six food items of the IFCDB identified as biodiverse plant foods, thirty-four were not considered when calculating the FAO Biodiversity Indicator, leading to a value of 52. Indeed, only biodiverse foods with certain descriptions on taxonomic rank below species are considered in the FAO indicator. This is likely due to FAO’s specific interest in promoting individual cultivars or varieties, while the current study is intended to identify any indication of biodiversity, even without identifying the individual variety or cultivar.

The FAO Biodiversity Indicator was also difficult to apply to the 2019 West African Food Database, the latest database published by FAO^(48)^. Even though this database is more comprehensive in respect to the 2012’s edition, food varieties are identified only by the common name. It is the case for staple foods such as maize and millet. Even when the variety is known, scientific names often remain limited to the species level, hindering an accurate assessment of the FAO Biodiversity Indicator.

However, the FAO Biodiversity Indicator calculated for the IFCDB is useful for comparative purposes. This indicator was assessed by Stadlmayr^(22)^ in the Australian and Thailand national FCDB reaching 536 and 319, respectively. The Stadlmayr’s study also reported the overall FAO Biodiversity Indicator across large world regions: considering the national FCDB available at that time for Italy, Greece and Sweden; a total of only seventy-four foods described as NUS, wild or below the species level were identified^(22)^. These results are difficult to compare with the findings from the current IFCDB since they are outdated.

### Conclusions

Through this study, the intrinsic biodiversity richness of the IFCDB at species level and below was enlightened. The implicit below the species’ information present in names was made explicit and easier to use for future studies. This suggests that in future database updates, it will be appropriate to always include the full scientific name for subspecies, varieties, and cultivars, where the Italian common name allows it. The negative implications – in terms of biodiversity richness of FCDB – of sampling methods used in the past in Italy and other countries (where more varieties/cultivars were blended before analysis) were highlighted. The biodiversity richness of the IFCDB could be further increased by performing analytical determinations of food components in more biodiverse food appropriately sampled and described. Since this activity is resource intensive, it will be essential to prioritise which food items to analyse.

One priority could be the analysis of varieties of plant species, which are largely consumed, such as rice and wheat to facilitate and promote the consumption of a wide range of biodiverse staple foods. This could significantly impact agrobiodiversity while increasing dietary diversity. The analysis of more NUS could also facilitate their promotion to reduce the risk of extinction of some crops and to benefit agrobiodiversity. For these reasons, this study can raise awareness among food system stakeholders, such as producers, policymakers and public health researchers about the impact of food biodiversity on public health.
